# Permanent Closure of the Jaws, with Report of a Case

**Published:** 1892-04

**Authors:** Wm. Knight

**Affiliations:** Prof. of Anatomy and Oral Surgery


### Permanent Closure of the Jaws, With Report of a Case.

BY WM. KNIGHT, M.D., D.D.S., PROF. OF ANATOMY AND ORAL SURGERY.

Read before the Mississippi Valley Association of Dental Surgeons, March 10, 1892.

The boundaries of the mouth consist of a very dilatable sac, lined by an elastic mucous membrane. Thus the walls of the cavity are prevented from being unduly stretched, or lapsing into folds when the mouth is closed. Of course any cause that may destroy any part of this lining membrane will interfere with its movements in proportion to the injury sustained. A mucous membrane, after recovering from an injury or ulcerative disease, is much more liable to the so-called secondary cicatrix—atrophy, than is the skin of the body. This atrophy is slow but irresistable in its progress, even when but small portions of the mucous membrane have been destroyed, there follows gradual but complete immobility of the jaws. Prof. Esmarch, in his valuable essay upon the subject, says: "Injuries to the mucous membrane of the cheek damage the mobility of the lower jaws in a greater or less degree by their cicatrizations, as is well known, and the cause of this ankylosis of the lower jaw, is often thought to be a growing together of the inner surface of the cheek with the bones or gums; this is not a correct view, however, and has in many cases led to improper treatment."

Permanent closure of the jaws is the result of many affections of the Tempero-Maxillary joints. It may be due to a severe sprain or concussion or arthritic inflammation, leading to a deposition of plastic matter and the conversion of this substance into cellulo-fibrous cartilaginous or osseous tissue. Again, permanent closure of the jaws may follow the exanthemata, as in the case of scarlet fever, reported by Dr. Mass, of Breslau; or be caused by pressure of a tumour in the parotid region, making a direct pressure upon the tempero-maxillary joint. Immobility of the jaw is occasioned in rare instances by an osseous bridge extending from the lower to the upper jaw or from the lower jaw to the temporal bone. Dr. Samuel Gross, writing upon this subject, says: "However induced, the effect is not only inconvenient, seriously interfering with mastication and articulation, but it is often followed, especially if it occur early in life, by a stunted development of the jaw, exhibiting itself in marked shortening of the chin and in an oblique direction of the front teeth." The treatment of this distressing condition has been until recent years most unsatisfactory. In cases of immobility from cicatricial tissue, experience has amply proved the utter uselessness of excising the constricted parts and resorting to after dilitation. Dr. Gross, noticing this, says: "When the immobility depends upon the presence of inodular tissue, the proper remedy is excision of the offending substance, an operation which is both tedious, painful and bloody, and unfortunately not followed by any but the most transient relief, owing to the tendency in the parts to reproduce the adhesions, however carefully and thoroughly they may have been removed. During my residence in Kentucky, I had a large share of such cases and although I never failed to make the most thorough work—not infrequently repeating the operation several times, at intervals of a few months—it is my duty to state that few of them were permanently relieved. After the excision is effected, the patient must make constant use of the wedge, wearing it for months and years, so as to counteract the tendency to reclosure". Prof. Esmarch, who has a large experience in these cases, writes as follows; "All the hitherto received methods, such as freeing or cutting through of the cicatrix from the mouth—the separation of the whole cheek—in order to accomplish this perfectly, the extirpation of the mass of cicatrix— the application of mechanical apparatus in order to drag the jaws asunder by degrees, and so can only be of avail in those cases where in some angle or other, there is found a remnant of mucous membrane. If one succeeds after separation of the cicatrix in preventing, by the application of mechanical means for a long time, the cicatrization in the undesirable direction—the contraction will take place in another direction and by degrees will drag the remnant of mucous membrane up to the skin. In every case, it takes years before such methods can be properly estimated; for, as far as is known, the secondary shrinking of a cicatrix takes place very late, even after complete or sufficient healing over has occurred. Putting aside the more favorable cases, there still remains a number of patients of this kind, in whom the usual methods produce no lasting cure, just because there is no more old mucous membrane left; and for these cases, I recommend the formation of an artificial joint in front of the contractions in order to give, at least the other half of the jaw some, although a limited motion, and so to lessen considerably the sufferings of these unfortunate patients." The suggestion of Prof. Esmarch, to form an artificial joint in front of the contraction has been productive of much good. Previously to this time, Dieffenbach had recommended, as well as tried, the formation of an artificial joint, but behind the contraction, and naturally without any good result; since the impediment to motion lies more forward and thus is not removed. This proposal of Prof. Esmarch to form a false joint in front of the cicatrix, was suggested to him by a case, which came under his care in 1854, in which considerable destruction of the cheek and contraction of the cicatrix had occurred, together with immobility of the jaw and necrosis of a portion of it. The necrosis was fortunately in front of the cicatrix. The bone having been removed, it was found that mobility was restored and a useful amount of movement obtained. Dr. Mitchell Henry was the first, to put Esmarch's suggestion into practice, his patient, however, dying from Pyæmia.

Christopher Heath, F. R. C. S., has since had several successful cases, so this operation is now an established one in surgery.

The operation suggested by Prof. Esmarch is especially indicated where only one side of the jaw is affected usually securing to the patient a useful, though one sided movement of the bone. Various modifications of this operation have since been devised and applied by different surgeons to suit special cases, and thus many persons afflicted with closure of the jaws have been relieved.

In order to give relief in cases of ankylosis due to destructive disease of the tempero-maxillary articulation, our treatment must be directed with the aim of securing a false joint, or joints in cases of double ankylosis, through some point of the ramus and not as some operators have done, in front of the masseter. This is sufficient to liberate the body of the bone so as to enable the patient to open the mouth, but the hand has to be brought into use in the act of masticating. If the immobility be due to fibrous adhesions formed between the condyle and glenoid cavity, the method as practiced by Mr. Spenton of dividing the adhesions with the tenomy knife is to be recommended. In cases of rheumatoid arthritis in which the suffering is great, as well as in cases of osseous ankylosis of the tempero-maxillary joint, excision of the condyle seems to offer the best means of giving relief. The operation through the ramus above the angle appears to me to be the most suitable in all cases of double ankylosis, not of rheumatic origin. Our aim being, in removing a small wedge of bone to establish an artificial joint; should the result prove successful, all of the muscles of mastication excepting the external Ptyregoid, have their functions retained to the benefit of the patient.

Rokitansky describes the unnatural joints resulting from fracture as of two kinds, "one, more or less resembling a synarthosis, the other, like a diarthrosis and accordingly, in its proper sense, a new joint. In the former case, the fractured ends of the bone are held together by a ligamentous tissue; either a disc of ligament, the thickness of which may vary, is interposed between them and allows of but little movement, or as occurs when there has been loss of substance from injury, absorption of fractured ends or otherwise—ligamentous bands connect the fragments and allow them to move freely on each other. The connecting tissue appears to be nothing more than the intermediate substance which has failed to become transformed into secondary callus and remains in its first state. In the second case, a ligamentous articular capsule is formed and is lined by a smooth membrane which secretes synovia.

The fractured surfaces adapt themselves to each other and become covered with a layer of tissue which is fibro-ligamentous or more or less fibro-cartilagenous. They may articulate immediately with one another or may have between them an intervening layer of ligament which corresponds to an articular cartilage and their movement upon each other is more or less free according to the size of the articular capsule and the form of the articulatory surfaces. These last are sometimes horizontal and smooth. They glide over each other and allow of restricted motion; sometimes one surface becomes convex and the other concave; sometimes both are rounded off and lying within a capacious capsule far apart; they come in contact only during particular movements. The articulating capsule is the product of the inflammation of the soft parts; the cartilaginous formed layer, which covers the ends of the bone is secondary callus, arrested in its metamorphosis and converted into a fibrous tissue. The other ligamentous cords which are sometimes present and the structure resembling an interarticulating cartilage are remnants of the intermediate substance. Both forms of new joint, but more particularly the synarthrodial form have an analogue in the lateral new joints sometimes formed between the masses of callus thrown around two adjoining bones."

Having thus reviewed the different modes of operating for permanent closure of the jaws, and the manner in which movement may be secured, I would like to read to you a case which has been brought before the Faculty of the Ohio College of Dental Surgery, in which the members during the past session, have shown great interest. I submit the entire case, as well as the details of the treatment to your criticism, while the patient herself is present for examination.

On September 30, 1891, Barbary Engel, aged eighteen, called at the consulting rooms, having been sent by Dr. H. A. Smith. An examination showed a pitiful condition of the mouth. There was no movement whatever in the lower jaw. The gums were swollen, red, soft, and bled upon the slightest touch; some of the incisors had been extracted but the remaining teeth, part of which were in a decayed condition, were firmly locked, and crowded one another from their normal position. From this miserable condition, she pleaded earnestly to be relieved. She has a clear, healthy complexion, a good development of head, bright, intelligent looking blue eyes. These characteristics give expression to a face, that owing to a marked recession of the chin, attending upon arrested development of the lower jaw, would otherwise have the stamp of idiocy.

At the age of three years she had a fall upon the chin, which was succeeded by swelling about the face and gradual tightening of the jaw, until complete closure ensued. From this time the jaw has remained immovable. Her nourishment, necessarily consisted entirely of liquids, to which most likely the clearness of her complexion is due.

Within the last fourteen years several attempts by different surgeons were made, under chloroform, to force open the mouth but without success, still, I thought something more should be attempted to relieve her sufferings. The various operations by which this condition may be remedied were carefully considered, and the one selected was, if possible, to form artificial joints above the angles on both sides of the lower jaw. The danger attending this and the uncertainty of success were explained to the patient as well as to her friends. The only promise given, being that in case of failing to liberate the jaw, we would extract the teeth.

On October 9th, assisted by Drs. H. A. Smith, H. Cundell-Juler, F. Kebler, H. C. Matlack, as well as the internes Drs. Newton and Scofield, in the presence of the Faculty and students of the Ohio College of Dental Surgery, the operation on the right side of the lower jaw was made. The incision passed below the ear along the posterior border of the Ramus to near the angle of the Maxilla; the anterior surface, as well as the border of the Ramus were then cleared of soft parts by the Periosteum and handle of the scapel, sufficiently to readily admit into the wound a small movable back saw; the bone was then carefully sawn through, the direction being obliquely upward from about a quarter of an inch above the angle. The attempt to remove a wedged-shaped piece of bone from the upper fragment with the saw, was not entirely successful, but with the aid of the bone cutting forceps and also the chisel and mallet, it was removed in small pieces. The hemorrhage throughout the operation was slight. The dental artery, by forcing and holding for a few seconds a small plug of soft wood into its canal, ceased bleeding. The soft parts were brought together and the wound dressed with iodoform. After the patient had been removed to her room, she did well; during the first forty-eight hours there was a slight rise of temperature and for a few days a little swelling of the neck as well as a difficulty in swallowing, but there was no movement of the jaw gained by this operation. The difficulty in swallowing was most likely due to the injury done to some of the fibres of the superior constrictor, as the attachment of this muscle to the jaw was involved in the portion of bone removed. The patient left the hospital on the eighth day after the operation.

The patient was again admitted to the hospital on October the 20th, and on the 23d was brought before the students of the Ohio College of Dental Surgery. An operation upon the left side of the jaw was then made, Drs. H. A. Smith, P. S. Connor, H. Cundell-Juler, Grant Mollyneaux and H. C. Matlack assisting. The operation in character was similar to the previous one and so liberated the jaw as to enable Dr. Mollyneaux to extract several badly decayed teeth that had caused the patient much suffering. From this operation, the symptoms were more pronounced, not only was the temperature higher but the swelling of the neck and the difficulty in swallowing were more severe; but these symptoms subsided after the third day. On the second day after the operation she was asked to protrude her tongue. To her surprise and great joy she found that she was able to do so. "Oh!" she exclaimed, "I can put my tongue out, I can put it out!" This organ, however, was very small being apparently arrested in its development from want of use; similarly as the inferior maxilla had been. Not an uninteresting feature in this case was the rapidity of growth which took place in her tongue, for within a week after she had first protruded this organ, it had gained double its size.

On the fourth day, Dr. Mollyneaux, assisted by Dr. G. C. Minturn, succeeded in obtaining, under chloroform inhalation, an impression of the interior of the mouth. The molar teeth were lying horizontally with the crowns facing the tongue, but with the use of mouth washes, first containing permanganate of potash, and later one more astringent containing tincture of hydrastis with chlorate of potash, her mouth was relieved of its foul condition and became clean and sweet. The gums and oral mucous membrane assumed a firm and healthy appearance. From the impression made of her mouth, silver shields were applied which have assisted in maintaining the movement which has been gained. Her speech, which before the operation was muffled, became clear and distinct. Her condition was thus decidedly improved, although but slight movement of the jaw had been gained. She left the hospital on the eighth day quite well, with the exception of a little healthy pus oozing from the wound on either side.

For some time subsequently, the patient was daily under my observation. Within a week of leaving the hospital, she was able to chew bread and eggs, and finally on November 9, 1891, for the first time since she was three years old, she was able to eat meat; a quail contributing to her meal.

The movement in her jaw is an up and down movement only, no lateral movement whatever being present. It is evident that in this case a double synarthrodial joint has been established. It would have been more satisfactory to have secured a double arthrodial joint.

There is not more than an eighth of an inch movement in the molar region, but this to the patient is valuable. The scars upon the face are not very noticeable and will become less so in time. The present condition of the patient is good, as the gentlemen present can see.

